# Multisite Atherosclerosis and SCORE2-Based Risk Stratification in Psoriatic Arthritis: A Phenotype-Dependent Role of Vascular Territories

**DOI:** 10.3390/biomedicines14061395

**Published:** 2026-06-20

**Authors:** Lilyan C. Charca, Ignacio Braña, Marta Loredo, Paula Alvarez, Estefanía Pardo, Stefanie Burger, Rubén Queiro

**Affiliations:** 1 Rheumatology Section, Fundació Hospital de l’Esperit Sant, 33011 Santa Coloma de Gramenet, Spain; lily.charca@gmail.com; 2 Rheumatology Division, Central University Hospital of Asturias, 33011 Oviedo, Spain; i.brana.abascal@hotmail.es (I.B.); mloredomart@gmail.com (M.L.); paulalvarez992@gmail.com (P.A.); estefaniapardoc@gmail.com (E.P.); stefanie.nam@gmail.com (S.B.); 3 Department of Medicine, Oviedo University School of Medicine, 33011 Oviedo, Spain; 4 Translational Immunology Division, Health Research Institute of the Principality of Asturias (ISPA), 33011 Oviedo, Spain

**Keywords:** carotid ultrasound, femoral ultrasound, subclinical atherosclerosis, cardiometabolic burden, cardiovascular risk, SCORE2, psoriatic arthritis

## Abstract

**Background:** Cardiovascular (CV) risk is increased in psoriatic arthritis (PsA), yet vascular assessment has largely focused on carotid arteries, potentially underestimating systemic atherosclerosis. **Objective:** The objective of this study was to characterize the distribution and concordance of atherosclerotic plaques across carotid, femoral, and aortic territories in PsA and evaluate their incremental value over SCORE2. **Methods:** In this cross-sectional study, 250 unselected patients with PsA underwent carotid and femoral ultrasound and abdominal X-ray. Plaque prevalence and multiterritorial involvement (≥2 vascular beds) were assessed. Agreement between territories was evaluated using Cohen’s κ. In patients aged 50–69 years, the incremental value of vascular territories over SCORE2 was evaluated using ROC curves, bootstrap-corrected decision curve analysis (DCA), and reclassification metrics (IDI and continuous NRI). **Results:** Plaques were detected in carotid (36.0%), femoral (62.8%), and aortic (31.6%) territories, with multiterritorial involvement in 43.2%. Agreement between vascular beds was moderate (κ ≈ 0.35). Notably, 48.1% of patients without carotid plaques had femoral involvement. SCORE2 categories showed a strong gradient with plaque prevalence (*p* < 0.0001). In patients aged 50–69 years, adding vascular imaging improved discrimination for multiterritorial disease (AUC 0.73 vs. 0.86–0.90). Reclassification analyses demonstrated that carotid plaque substantially improved the identification of multiterritorial atherosclerosis (IDI 0.32, 95% CI 0.18–0.50; continuous NRI 1.33, 95% CI 1.08–1.60), with similar results observed for aortic plaque (IDI 0.33, 95% CI 0.20–0.50; continuous NRI 1.24, 95% CI 0.99–1.48). Femoral plaque provided a more modest improvement (IDI 0.26, 95% CI 0.16–0.37; continuous NRI 1.11, 95% CI 0.80–1.33). Conversely, when the outcome was defined as the presence of any plaque, femoral plaque provided the greatest incremental value over SCORE2 (AUC 0.96, 95% CI 0.93–0.99). Bootstrap-corrected DCA confirmed improved net benefit. **Conclusions:** The incremental value of vascular imaging over SCORE2 appears to be phenotype-dependent. Femoral plaque provided the greatest improvement for detecting the presence of subclinical atherosclerosis, whereas carotid and aortic plaques offered greater incremental value for identifying multiterritorial vascular involvement. These findings support a tailored, multiterritorial approach to cardiovascular risk assessment in patients with PsA.

## 1. Introduction

Psoriatic arthritis (PsA) is a chronic inflammatory disease that affects up to one-third of patients with psoriasis and is characterized by a heterogeneous clinical spectrum involving peripheral arthritis, axial disease, enthesitis, and dactylitis. In recent years, PsA has increasingly been recognized as a systemic disease, extending beyond joint and skin manifestations to include important comorbidities, among which cardiovascular (CV) disease is particularly relevant [1,2]. A growing body of evidence indicates that patients with PsA have an increased risk of myocardial infarction, stroke, and CV mortality compared with the general population. This excess burden is partly attributable to an increased prevalence of traditional CV risk factors (CVRFs) such as hypertension, obesity, dyslipidemia, and smoking, but also to chronic systemic inflammation, which promotes endothelial dysfunction, arterial stiffness, and accelerated atherosclerosis [1,2].

Non-invasive imaging has become a cornerstone for detecting subclinical atherosclerosis in immune-mediated diseases. Carotid ultrasound has been widely used to assess intima–media thickness (IMT) and detect carotid plaques, both of which correlate with future CV events [3]. In patients with rheumatoid arthritis and PsA, the presence of carotid plaques has been shown to reclassify a substantial proportion of individuals from low or moderate risk to high risk according to conventional scoring systems [2,4]. However, atherosclerosis is a systemic disease that may affect different arterial beds with varying patterns and timing. Restricting the assessment to the carotid arteries may therefore underestimate the true burden of vascular disease in PsA.

Emerging evidence from studies in the general population and in selected cohorts of patients with autoimmune diseases suggests that femoral and aortic territories may provide complementary information [5,6,7]. Femoral plaques have been described as highly prevalent and may appear earlier than carotid lesions [8]. Similarly, abdominal aortic X ray can capture additional structural damage and identify patients with advanced or diffuse atherosclerosis [7]. Yet, to date, very few studies in PsA have systematically examined vascular territories beyond the carotid arteries, and none have comprehensively assessed the degree of concordance between different arterial sites. Understanding whether involvement in one vascular bed predicts disease in another is crucial for optimizing screening strategies and determining the incremental value of extending imaging protocols.

Another important aspect is the integration of imaging data with clinical risk prediction models. The SCORE2 algorithm, recently recommended by European guidelines to replace the classic SCORE, has refined CV risk assessment by incorporating age-specific thresholds and updated epidemiological data [9]. Nevertheless, its performance in PsA remains uncertain, and it is unknown to what extent SCORE2 categories correlate with the actual presence and distribution of structural atherosclerosis across multiple vascular beds. Bridging this gap could help to identify patients at high risk of CV events who might benefit from more aggressive preventive strategies.

In this context, we designed the present study to provide a comprehensive characterization of structural atherosclerosis in PsA. Specifically, our objectives were: (i) to determine the prevalence of plaques in the carotid, femoral, and aortic territories; (ii) to evaluate the concordance between arterial beds; (iii) to identify independent clinical and disease-related correlates of plaque presence; and (iv) to examine the relationship between SCORE2 categories and multisite structural atherosclerosis. This integrative approach seeks to clarify the true extent of vascular disease in PsA and its interplay with contemporary risk prediction tools.

## 2. Materials and Methods

### 2.1. Study Design and Participants

We conducted a multicenter cross-sectional study including PsA cohorts from two tertiary care centers. Eligible participants were adults (≥18 years) with a diagnosis of PsA established by the treating rheumatologist according to the CASPAR criteria [10]. Patients with prior CV disease were not excluded; instead, they were classified as very-high risk according to SCORE2 to preserve external validity. Patients were recruited consecutively from established PsA outpatient clinics at both participating tertiary-care centers during routine clinical follow-up visits. No advertisement-based recruitment was used. The study was approved by the local Ethics Committee of Principality of Asturias (protocol number 2020.423). All patients provided written informed consent.

### 2.2. Clinical and Disease-Related Variables

Clinical data were obtained from standardized rheumatology assessments and electronic medical records. Disease activity, disease impact, and treatment exposure were recorded at the study visit. Demographic variables included age, sex, and body mass index (BMI, kg/m^2^). Lifestyle factors were recorded as smoking status (never, former, or current), later collapsed into ever- vs. never-smoker. Disease-related characteristics included duration of psoriasis and PsA, as well as the presence of structural joint damage defined as radiographic erosions. Disease activity was assessed with the Disease Activity in Psoriatic Arthritis (DAPSA), dichotomized as remission/low vs. moderate/high activity. Disease impact was measured with the Psoriatic Arthritis Impact of Disease questionnaire (PsAID-12), defining high impact as PsAID ≥ 4. Exposure to biologic therapy was coded as present or absent at the time of vascular assessment. Classical CVRFs (hypertension, dyslipidemia, diabetes, obesity, hyperuricemia) were also recorded. A variable termed “any CVRF” was generated. It was defined as the presence of at least one of the following: hypertension, diabetes mellitus, dyslipidemia, obesity, or hyperuricemia. Hypertension was defined as a prior physician diagnosis, use of antihypertensive medication, or both.

### 2.3. Cardiovascular Risk Stratification

CV risk was estimated using the SCORE2 algorithm, in line with European Society of Cardiology recommendations [9]. Three age-specific modalities were applied: <50 years: low, moderate, high risk; 50–69 years: low–moderate, high, very high risk; ≥70 years: moderate, high, very high risk. For global analyses, a harmonized SCORE2 variable (0–3) was derived to assign a single category to each patient, while stratified analyses were also performed by age group.

### 2.4. Assessment of Structural Atherosclerosis

All patients underwent high-resolution B-mode ultrasound of the carotid arteries (common, bifurcation, and internal) and the common femoral arteries (including bifurcation). Abdominal aortic calcification was assessed by plain abdominal X ray. Plaques were defined according to the Mannheim consensus as a focal structure encroaching into the arterial lumen ≥ 0.5 mm or >50% of the surrounding intima–media thickness, or with a thickness ≥ 1.5 mm measured from the media–adventitia interface to the intima–lumen interface [11]. For each territory (carotid, femoral, aortic), plaque was coded as a binary variable. Multisite involvement was defined as the presence of plaques in ≥2 vascular territories.

### 2.5. Statistical Analysis

All analyses were two-sided with α = 0.05. Descriptive statistics were presented as mean (SD) or median (IQR) for continuous variables and counts (%) for categorical variables. Prevalence of plaque was reported overall and stratified by sex and age quartiles. We compared plaque prevalence by sex using chi-square (Fisher’s exact if needed), and across age quartiles using chi-square. For age quartiles we additionally performed pairwise 2 × 2 comparisons with Bonferroni correction (six contrasts per outcome). We quantified agreement using Cohen’s κ with 2 × 2 contingency tables for each pair of beds (carotid vs. femoral, carotid vs. aortic, femoral vs. aortic). κ values were interpreted in the usual manner. We also evaluated the overlap between vascular territories using conditional probabilities and joint distributions. For each index territory (carotid, femoral, or aortic), we calculated the probability of plaque in the other territories conditional on the presence of plaque in the index. In addition, we estimated the probability of simultaneous involvement of both remaining territories. A joint distribution table was generated to describe the frequency of all possible plaque combinations. This approach allows quantification of concordance patterns and highlights the diagnostic yield of examining multiple vascular beds beyond the carotid arteries alone. SCORE2 cardiovascular risk categories were derived according to age group using the dedicated algorithms: <50 years (categories 0–2, corresponding to low, moderate, and high risk), 50–69 years (categories 1–3, low–moderate, high, and very high risk), and ≥70 years (older people, OP; categories 1–3, moderate, high, and very high risk). A unified SCORE2 variable was created to harmonize categories across age strata. Prevalence of carotid, femoral, aortic, and multisite (≥2 territories) plaques was calculated within each SCORE2 category. Differences across categories were assessed by chi-square tests for trend. In addition, subgroup analyses were performed separately for each age stratum (<50, 50–69, and ≥70 years). For each outcome (carotid, femoral, aortic, multisite) we fitted logistic regression with the same a priori covariate set (no stepwise selection): sex, age, BMI, psoriasis duration, PsA duration, any-smoking, any CVRF, erosions, DAPSA moderate–high, PsAID ≥ 4, biologic exposure (yes/no). We report adjusted odds ratios (ORs) with 95% CIs and *p*-values. Linearity of continuous covariates in the logit was inspected; given approximately linear relationships and to preserve power, continuous forms were retained. Multicollinearity was assessed via variance-inflation factors (VIF), targeting VIF < 5. Model fit and residual diagnostics were reviewed. Given outcome frequencies and the number of parameters, events-per-variable thresholds were adequate (complete-case n = 224; event counts supported EPV ≥ 10 in all models). Missingness was low to moderate across predictors; multivariable analyses used complete-case datasets (no imputation). We verified that the complete-case sample remained representative of the full cohort on key demographics and outcomes.

In a secondary analysis, we evaluated the incremental value of vascular territories over SCORE2 for the identification of multiterritorial atherosclerosis (defined as plaque in ≥2 vascular beds). Analyses were restricted to patients aged 50–69 years with available SCORE2 data (n = 131), representing the subgroup in which SCORE2-based risk stratification is most clinically actionable. Logistic regression models were constructed using SCORE2 alone and in combination with carotid, femoral, or aortic plaque. Discriminative performance was assessed using receiver operating characteristic (ROC) curves and area under the curve (AUC). Clinical utility was evaluated using decision curve analysis (DCA) across threshold probabilities of 5–40%. To account for model optimism, DCA was internally validated using bootstrap resampling (1000 iterations), with models trained in bootstrap samples and evaluated in the original dataset. To further assess the incremental value of vascular imaging over SCORE2, reclassification analyses were performed in the subgroup of patients aged 50–69 years. Improvements in risk prediction were quantified using the integrated discrimination improvement (IDI) and the continuous net reclassification improvement (NRI). The continuous NRI was calculated by assessing the proportion of individuals with events who were assigned a higher predicted probability and the proportion of individuals without events who were assigned a lower predicted probability after adding each vascular territory to the base SCORE2 model. IDI was defined as the difference in discrimination slopes between models. These analyses were performed using predicted probabilities derived from logistic regression models.

Analyses and figures were produced in Python 3.11 using pandas, statsmodels, SciPy, and matplotlib.

## 3. Results

### 3.1. Study Population

A total of 250 patients with PsA were included. The median age was 63.2 (IQR: 52.6–69.6) years, and 119 (47.6%) were women. The median duration of psoriasis and PsA was 24.0 (IQR: 14.0–39.5) and 11.0 (IQR: 7.0–13.0) years, respectively. Traditional CVRFs were frequent: 89 (35.6%) had hypertension, 169 (67.6%) dyslipidemia, 56 (22.4%) obesity, 127 (50.8%) were ever-smokers, and 25 (10.0%) patients were diabetics. Overall, 177 (70.8%) patients were receiving biologic therapy at the time of vascular assessment. Table 1 summarizes disease characteristics of the study cohort.

### 3.2. Prevalence and Distribution of Atherosclerotic Plaque

**Figure 1:**
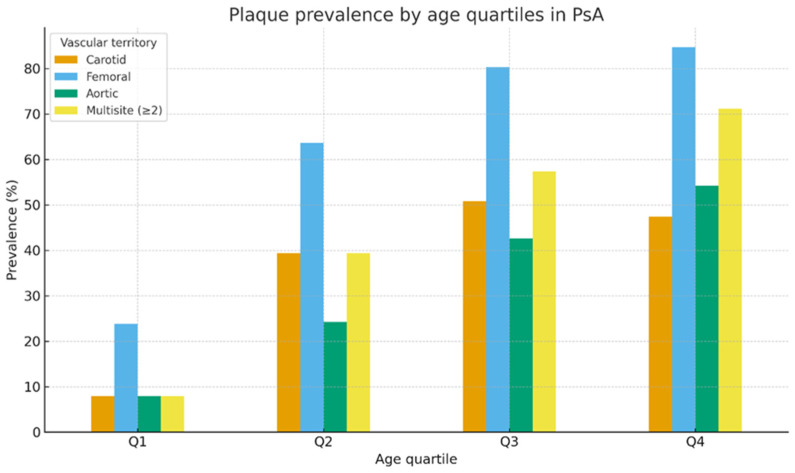
Plaque prevalence by age quartiles in patients with psoriatic arthritis. Bars represent the proportion of patients with carotid, femoral, aortic, and multisite (≥2 territories) atherosclerotic plaques across age quartiles (Q1–Q4). A clear age-related gradient was observed in all vascular territories, particularly in femoral and multisite involvement (overall *p* < 0.0001 for all comparisons across quartiles; Bonferroni-adjusted pairwise tests significant for Q1 vs. Q2–Q4). Age quartiles were defined as follows: Q1 (≤51.9 years), Q2 (>51.9 to ≤62.6 years), Q3 (>62.6 to ≤68.9 years), and Q4 (>68.9 years).

Structural atherosclerosis was highly prevalent. Femoral plaques were the most frequent across the cohort, followed by carotid and aortic involvement. Overall, carotid plaques were detected in 90 (36.0%) patients, femoral plaques in 157 (62.8%), and aortic plaques in 79 (31.6%). Multisite involvement (plaques in ≥2 territories) was present in 108 (43.2%) patients. When stratified by sex, men showed significantly higher prevalence of femoral plaques compared with women (72.5% vs. 52.1%, *p* = 0.001). Smaller, non-significant sex differences were observed for carotid and aortic plaques. By age quartiles, a clear gradient was observed for all territories (*p* for trend <0.0001), with multisite involvement reaching >70% in the oldest quartile (Figure 1). Pairwise comparisons confirmed significant differences between the youngest quartile (Q1) and the remaining strata after Bonferroni correction.

### 3.3. Agreement Between Vascular Territories

Agreement between vascular beds was moderate: carotid and femoral plaques (κ = 0.35), femoral and aortic (κ = 0.36), and carotid and aortic (κ = 0.35). However, conditional probability analyses revealed that among patients with carotid plaques, 88.9% also had femoral involvement and 53.3% had aortic involvement; conversely, 48.1% and 19.4% of patients without carotid plaques still had femoral and aortic plaques, respectively. Joint distribution analysis demonstrated that the most frequent patterns were femoral-only involvement (20.0%), carotid–femoral (13.2%), and plaques in all three territories (18.8%). Isolated aortic plaque was rare (1.6%) (See Supplementary Material S1).

### 3.4. Clinical Correlations of Plaque Localization

In multivariable logistic regression (N = 224), several independent associations emerged. Carotid plaques were independently associated with older age (OR 1.11 per year, 95% CI 1.07–1.16), smoking (OR 2.55, 95% CI 1.27–5.12), the presence of CVRFs (OR 3.18, 95% CI 1.04–9.69), erosive disease (OR 5.81, 95% CI 2.31–14.59), PsAID ≥ 4 (OR 2.36, 95% CI 1.07–5.19), and biologic exposure (OR 5.69, 95% CI 2.37–13.63). Female sex was negatively associated with carotid plaque (OR 0.48, 95% CI 0.24–0.98). Femoral plaques were associated with older age (OR 1.16 per year, 95% CI 1.10–1.21), male sex (OR 0.14 for women, 95% CI 0.05–0.35), longer arthritis duration (OR 1.12 per year, 95% CI 1.04–1.21), higher BMI (OR 1.12, 95% CI 1.01–1.23), the presence of CVRFs (OR 6.91, 95% CI 2.20–21.71), and moderate-to-high disease activity according to DAPSA (OR 3.73, 95% CI 1.35–10.33). Smoking showed a borderline association. Aortic plaques were independently associated with older age (OR 1.09 per year, 95% CI 1.05–1.14), smoking (OR 2.29, 95% CI 1.15–4.56), and the presence of cardiovascular risk factors (OR 9.51, 95% CI 1.98–45.69). Moderate-to-high DAPSA and biologic exposure showed borderline associations. Multisite involvement was independently associated with older age (OR 1.17 per year, 95% CI 1.12–1.23), smoking (OR 2.24, 95% CI 1.11–4.52), erosive disease (OR 4.27, 95% CI 1.60–11.43), and biologic exposure (OR 4.46, 95% CI 1.86–10.70). Female sex was negatively associated with multisite disease (OR 0.32, 95% CI 0.15–0.69).

Together, these models suggest that femoral and aortic plaques are predominantly associated with traditional cardiovascular risk factors, whereas carotid and multisite involvement additionally reflect disease-related factors, including structural damage, disease impact, smoking exposure, and treatment intensity (see full models and forest plots in Supplementary Material S2).

### 3.5. Relationship Between SCORE2 Categories and Plaque Involvement

**Figure 2:**
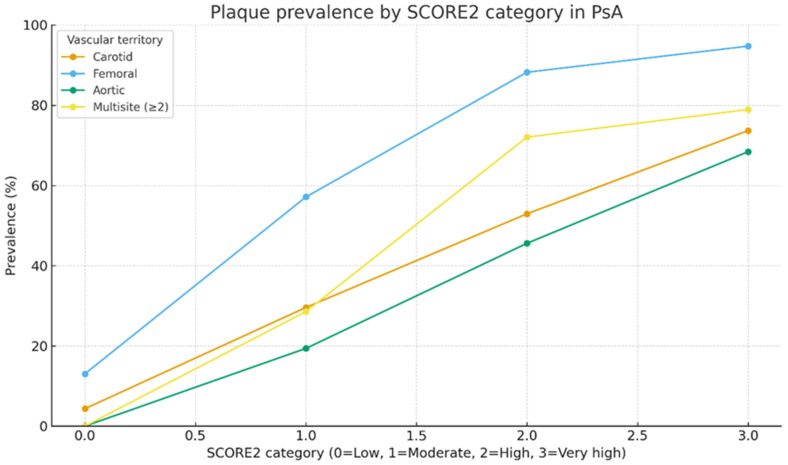
Risk gradient between plaque prevalence and SCORE2 risk estimates. This line chart shows how prevalence of carotid, femoral, aortic, and multisite plaques increases stepwise from low SCORE2 (0) to very high SCORE2 (3).

SCORE2 categories were available for 231 patients (19 missing). Distribution was as follows: 0 (low) in 46 patients, 1 (moderate) in 98, 2 (high) in 68, and 3 (very high) in 19. A clear risk gradient was evident (Figure 2): Carotid plaques: 4.3% (low), 29.6% (moderate), 52.9% (high), 73.7% (very high); femoral plaques: 13.0%, 57.1%, 88.2%, 94.7%; aortic plaques: 0%, 19.4%, 45.6%, 68.4%; and multisite involvement: 0%, 28.6%, 72.1%, 78.9%. All associations were significant (*p* for trend <0.0001).

Stratified analyses by age strata revealed consistent findings. In patients < 50 years, femoral plaques were already frequent at moderate risk. In the 50–69 group, the gradient was steep, with multisite involvement predominating at high and very high risk. In patients ≥ 70, plaque was nearly universal in high and very high categories, with multisite disease exceeding 70% (see Supplementary Material S3).

### 3.6. Incremental Value of Vascular Territories over SCORE2 (50–69 Years)

**Figure 3:**
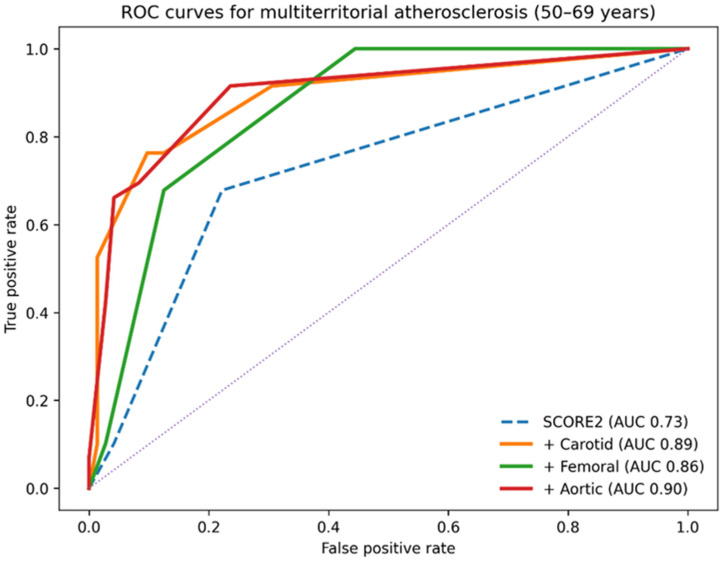
Discrimination performance of SCORE2 against vascular imaging for multisite atherosclerosis. Receiver operating characteristic (ROC) curves for the detection of multiterritorial atherosclerosis in patients aged 50–69 years. The addition of vascular imaging improved discrimination compared with SCORE2 alone, with similar performance observed for carotid and aortic plaque and slightly lower performance for femoral plaque.

Among patients aged 50–69 years with available SCORE2 data (n = 131), SCORE2 alone showed moderate discrimination for multiterritorial atherosclerosis (AUC 0.73, 95% CI 0.65–0.80). The addition of vascular imaging improved discrimination, particularly for carotid plaque (AUC 0.89, 95% CI 0.83–0.94) and aortic plaque (AUC 0.90, 95% CI 0.84–0.94), with slightly lower performance for femoral plaque (AUC 0.86, 95% CI 0.80–0.92). Figure 3 shows these findings.

**Figure 4:**
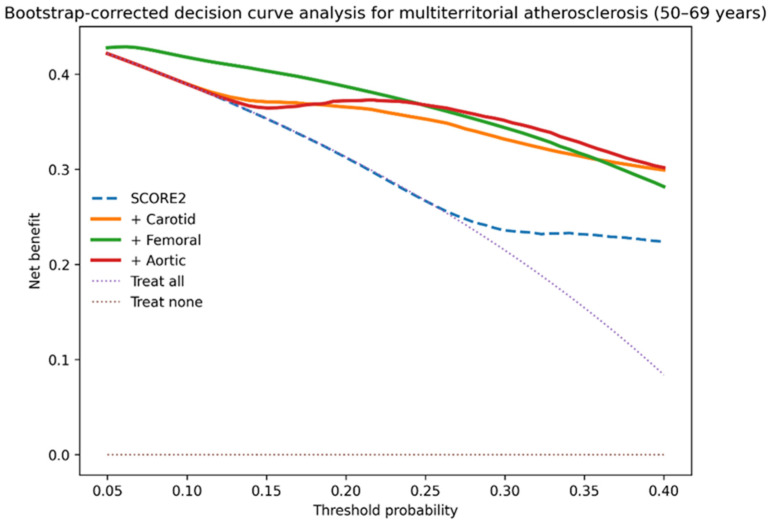
Decision curve analysis (DCA) for multisite atherosclerosis. Bootstrap-corrected decision curve analysis for the detection of multiterritorial atherosclerosis in patients aged 50–69 years. The addition of vascular imaging to SCORE2 improved net benefit across clinically relevant threshold probabilities. Differences between vascular territories were modest, with broadly overlapping performance across most thresholds.

Reclassification analyses yielded consistent findings. The addition of carotid plaque substantially improved risk classification for multiterritorial atherosclerosis (IDI 0.32, 95% CI 0.18–0.50; continuous NRI 1.33, 95% CI 1.08–1.60). Similar results were observed for aortic plaque (IDI 0.33, 95% CI 0.20–0.50; continuous NRI 1.24, 95% CI 0.99–1.48), whereas the improvement associated with femoral plaque was more modest (IDI 0.26, 95% CI 0.16–0.37; continuous NRI 1.11, 95% CI 0.80–1.33). These findings suggest that carotid and aortic territories contribute more strongly to the identification of multiterritorial vascular involvement beyond SCORE2. In contrast, when the outcome was defined as the presence of any plaque (≥1 vascular territory), femoral plaque provided the greatest incremental value over SCORE2 (AUC 0.96, 95% CI 0.93–0.99), outperforming carotid plaque (AUC 0.83, 95% CI 0.76–0.88) and aortic plaque (AUC 0.80, 95% CI 0.72–0.85). Consistent findings were observed for both IDI and continuous NRI, supporting the utility of femoral imaging for detecting the presence of subclinical atherosclerosis. Bootstrap-corrected decision curve analysis confirmed that adding vascular imaging to SCORE2 improved net benefit across clinically relevant threshold probabilities. Differences between vascular territories were modest, with broadly overlapping performance across most thresholds (Figure 4).

## 4. Discussion

In this comprehensive evaluation of structural atherosclerosis in PsA, we demonstrate that vascular involvement is both common and heterogeneous across arterial territories. Femoral plaques emerged as the most frequent manifestation, present in more than half of patients even at moderate SCORE2 risk, and they frequently preceded carotid and aortic involvement. Carotid plaques, the traditional focus of vascular ultrasound in immune-mediated diseases, substantially underestimated the systemic burden, as nearly half of patients without carotid plaques still had femoral lesions. Multisite involvement was present in 43.2% of the cohort and became the dominant phenotype in older patients and in those classified at high or very-high SCORE2 risk.

A central novelty of our study is the systematic assessment of three vascular beds—carotid, femoral, and aortic—in a large PsA cohort, combined with an evaluation of their concordance, clinical correlates, and relationship with SCORE2 categories. To our knowledge, no previous PsA study has comprehensively addressed these issues. While carotid imaging has been extensively investigated in rheumatic diseases, femoral ultrasound and aortic evaluation have been largely overlooked. Our findings show that restricting the assessment to the carotid arteries alone leads to a substantial underestimation of systemic vascular disease. This reinforces the concept that PsA is characterized by diffuse vascular involvement and highlights the incremental diagnostic yield of extending vascular screening protocols.

Our multivariable analyses highlight that different vascular territories may reflect distinct pathophysiological drivers. Femoral and aortic plaques were predominantly associated with age and conventional CVRFs, while femoral involvement additionally showed associations with BMI, disease duration, and disease activity. In contrast, carotid and multisite plaques were additionally linked to structural joint damage (radiographic erosions), patient-reported disease impact (PsAID ≥ 4), and exposure to biologic therapies.

The association with erosions suggests that cumulative inflammatory burden and irreversible musculoskeletal damage may parallel vascular injury [12,13,14,15]. In this scenario, erosion serves not only as a marker of joint severity but also as a proxy for systemic tissue damage and chronic inflammation, both of which may accelerate vascular pathology. The link with high PsAID scores supports the notion that patients perceiving greater disease impact may also carry higher vascular risk, possibly through more severe inflammation or comorbid symptoms such as pain, fatigue, and reduced physical activity.

The observed association between biologic exposure and plaque, particularly for carotid and multisite outcomes, warrants careful interpretation. Rather than implying a deleterious effect of biologic therapy, this finding most likely reflects confounding by indication: patients with long-standing, severe, or refractory disease are more likely to have been exposed to multiple biologics and at the same time to develop irreversible joint and vascular damage. This interpretation aligns with prior observations in rheumatoid arthritis and PsA, where the most severely affected patients, often treated with biologics, carry the greatest cumulative inflammatory burden [16,17,18]. Longitudinal data are needed to clarify whether biologic therapy exerts neutral, protective, or adverse effects on vascular outcomes in PsA.

The excess CV risk in PsA is well established, with epidemiological data showing increased rates of myocardial infarction, stroke, and premature CV mortality compared with the general population [2,18,19]. However, most imaging studies in PsA and other immune-mediated diseases have relied on carotid ultrasound, following paradigms established in rheumatoid arthritis. Several groups have confirmed that carotid plaques are prevalent in PsA and confer additive prognostic information beyond traditional risk scores [20]. Our study expands this view by including femoral and aortic territories, revealing a broader and earlier vascular burden that would have been missed by carotid-focused assessment alone.

Evidence from non-rheumatic populations, such as the PESA study, has demonstrated that femoral plaques may appear earlier than carotid lesions and are closely related to metabolic risk factors [21]. Our findings in PsA parallel these observations, underscoring that femoral ultrasound captures a large proportion of patients with subclinical disease, many of whom would otherwise be misclassified as low or moderate risk based on carotid imaging or traditional scores. Furthermore, recent evidence suggests that femoral vascular ultrasound may be more cost-effective than carotid examination in estimating subclinical atherosclerosis in PsA [22].

Regarding aortic involvement, previous reports in systemic autoimmune diseases have shown variable prevalence, often linked to long disease duration, smoking, and metabolic risk [23,24,25]. In our cohort, aortic plaques were less frequent than femoral but showed strong associations with age, smoking, and composite CVRFs. Importantly, aortic plaques showed a borderline association with moderate-to-high DAPSA activity, whereas femoral plaques were significantly associated with higher disease activity, suggesting that persistent inflammation may contribute to vascular injury in selected arterial territories.

Another key contribution of this study is the validation of SCORE2 in PsA against multisite vascular imaging, representing the first such evaluation in this disease. Our findings demonstrated a clear and consistent gradient of plaque prevalence across SCORE2 categories, with strong statistical significance (*p* for trend <0.0001). This validates the clinical relevance of SCORE2 in PsA, extending prior data generated in rheumatoid arthritis cohorts [26].

At the same time, our study highlights important nuances. In patients under 50 years, femoral plaques were already frequent at moderate SCORE2 risk, indicating that SCORE2 may underestimate early subclinical disease in younger individuals with PsA. Conversely, in patients aged ≥70 years, plaque was nearly universal in high and very-high categories, illustrating a ceiling effect whereby SCORE2 categories provide little discrimination at older ages. These observations support the use of SCORE2 as a valid tool in PsA but also emphasize the added value of incorporating multisite imaging, particularly femoral ultrasound, to refine risk stratification in younger or moderate-risk patients.

Taken together, our findings align with growing recognition that conventional risk algorithms may underperform in immune-mediated diseases, particularly among younger patients with high inflammatory burden but fewer traditional risk factors [4,18,19]. Integrating SCORE2 with multisite imaging may therefore provide a more precise framework for personalized CV prevention in PsA.

A secondary analysis focusing on patients aged 50–69 years further refined these findings. In this clinically relevant subgroup, the incremental value of vascular imaging over SCORE2 was evident, although it differed according to the vascular phenotype assessed. Carotid and aortic plaque provided the greatest improvement for identifying multiterritorial involvement, a surrogate of more advanced and systemic disease, whereas femoral plaque showed superior performance for detecting any subclinical atherosclerosis. These findings were further supported by reclassification analyses, which showed greater improvement in both IDI and NRI for carotid and aortic plaque compared with femoral plaque.

These results support a complementary interpretation of vascular territories. Rather than a single optimal site, different arterial beds appear to capture distinct stages of vascular disease, with femoral involvement reflecting earlier cardiometabolic-driven atherosclerosis and carotid or aortic plaque indicating more diffuse and cumulative vascular burden. This phenotype-dependent effect reinforces the limitations of a purely carotid-centered approach and supports a more tailored strategy for CV risk assessment in PsA.

The implications of our findings for clinical practice are substantial. First, reliance on carotid ultrasound alone may substantially underestimate systemic atherosclerosis in PsA. Incorporating femoral ultrasound into vascular screening protocols could significantly increase sensitivity for detecting subclinical disease. This may be particularly relevant in patients with moderate SCORE2 risk or in those with discordant clinical features, such as severe joint damage or high patient-reported impact. Second, the strong associations between traditional CVRFs and femoral/aortic plaques underscore the need for systematic screening and aggressive management of these factors in PsA. Rheumatologists should actively monitor and control blood pressure, lipids, glycemia, smoking, and obesity, integrating CV prevention into routine PsA care. Our data also reinforce the importance of BMI as a modifiable contributor to vascular burden. Third, the associations of carotid and multisite plaques with erosions, PsAID, and biologic exposure suggest that musculoskeletal severity and patient-perceived disease impact may serve as clinical red flags for higher vascular risk. In practice, this means that PsA patients with more severe articular damage or higher disease impact scores may warrant intensified CV evaluation, including multisite imaging, even if their SCORE2 category appears only moderate.

Strengths of our study include the relatively large PsA cohort, the comprehensive assessment of three vascular territories, and the integration of clinical, radiographic, and patient-reported outcomes. The use of SCORE2, rather than the obsolete classic SCORE, enhances the contemporary relevance of our findings. Furthermore, the analytic approach—combining prevalence, concordance, conditional probabilities, and regression—provides a nuanced understanding of vascular heterogeneity in PsA. Limitations must also be acknowledged. The cross-sectional design precludes causal inference, and prospective studies are needed to determine whether multisite plaques predict future CV events in PsA. Imaging was performed at a single time point, so we did not assess plaque progression. The use of abdominal radiography detects calcified but not soft aortic plaques, potentially underestimating the true aortic burden. Because participants were recruited from tertiary referral centers, the proportion of patients receiving biologic therapies was relatively high. Consequently, the cohort may overrepresent patients with more severe or treatment-refractory disease, potentially limiting generalizability to community-based PsA populations. Finally, although major confounders were adjusted for, residual confounding by unmeasured factors (e.g., diet, physical activity, cumulative medication exposure) cannot be excluded.

Future research should address whether femoral and aortic plaques provide incremental prognostic value for hard CV outcomes such as myocardial infarction, stroke, and CV mortality in PsA. Prospective cohorts with linkage to event data will be crucial to confirm the predictive utility of multisite imaging. Finally, exploring whether different therapeutic approaches (e.g., IL-17 or JAK inhibitors) influence vascular outcomes could provide insight into the vascular benefits of modern PsA treatments.

## 5. Conclusions

Psoriatic arthritis exhibits a heterogeneous, multiterritorial pattern of atherosclerosis that is not fully captured by carotid imaging alone. The added value of vascular imaging over SCORE2 is phenotype-dependent, with femoral plaque improving early detection and carotid and aortic plaque identifying more advanced, multiterritorial disease. These results support a tailored approach to cardiovascular risk assessment in PsA.

## Figures and Tables

**Table 1:** Disease features of the study population and sex-related differences.

Disease Feature	Total n: 250	Men n: 131	Women n: 119	*p*-Value
Age, yrs, median (IQR)	63.2 (52.6–69.6)	62.6 (52.6–71.5)	63.9 (52.7–69.1)	NS
Psoriasis duration, yrs, median (IQR)	24.0 (14.0–39.5)	23.0 (12.0–38.0)	25.0 (14.0–41.5)	NS
PsA duration, yrs, median (IQR)	11.0 (7.0–13.0)	10.0 (7.0–13.5)	11.0 (8.0–13.0)	NS
BMI, median (IQR)	27.2 (25.0–29.7)	27.8 (25.5–29.7)	26.7 (24.0–29.6)	NS
Systolic blood pressure, mm/Hg, median (IQR)	123.0 (117.0–130.0)	125.0 (120.0–130.0)	120.0 [115.0–127.0)	0.001
Cholesterol mg/dL, median (IQR)	195.0 (168.0–227.5)	191.0 (164.5–222.5)	200.0 [170.0–234.5)	NS
HDL-cholesterol, mg/dL, median (IQR)	54.5 (46.0–63.0)	51.00 (42.0–61.0)	57.0 (51.0–66.0)	<0.001
HAQ, median (IQR)	0.75 (0.25–1.12)	0.62 (0.00–1.06)	0.87 (0.50–1.12)	0.006
PSAID12, median (IQR)	3.73 (1.21–5.24)	3.50 (1.07–5.40)	3.95 (2.03–4.93)	NS
cIMT, mm, median (IQR)	0.76 (0.67–0.90)	0.78 (0.68–0.95)	0.76 (0.65–0.90)	NS
Psoriasis family history, n (%)	123 (49.2)	71 (54.2)	52 (43.7)	NS
Oligoarthritis, n (%)	115 (46.0)	59 (45.0)	56 (47.1)	NS
Mixed, n (%)	80 (32.0)	55 (42.0)	25 (21.0)	<0.001
Polyarthritis, n (%)	47 (18.8)	15 (11.5)	32 (26.9)	<0.001
Axial, n (%)	8 (3.2)	2 (1.5)	6 (5.0)	NS
DIP disease, n (%)	37 (14.8)	21 (16.0)	16 (13.4)	NS
Dactylitis, n (%)	93 (37.2)	43 (32.8)	50 (42.0)	NS
Enthesitis, n (%)	83 (33.2)	53 (40.5)	30 (25.2)	0.015
IBD, n (%)	9 (3.6)	5 (3.8)	4 (3.4)	NS
Uveitis, n (%)	8 (3.2)	7 (5.3)	1 (0.8)	NS
Former smokers, n (%)	73 (29.2)	46 (35.1)	27 (22.7)	NS
Current smokers, n (%)	54 (21.6)	27 (20.6)	27 (22.7)	NS
CVD, n (%)	12 (4.8)	9 (6.9)	3 (2.5)	NS
Erosive disease, n (%)	55 (22.0)	30 (22.9)	25 (21.0)	NS
Mild BSA (≤3%), n (%)	112 (44.8)	59 (45.0)	53 (44.5)	NS
Moderate-severe BSA (>3%), n (%)	15 (6.0)	13 (9.9)	2 (1.7%)	0.044
Nail disease, n (%)	123 (49.2)	65 (49.6)	58 (48.7)	NS
Biologics, n (%)	177 (70.8)	89 (67.9)	88 (73.9)	NS
Rem-low DAPSA, n (%)	172 (68.8)	101 (77.1)	71 (59.7)	0.007
Moderate-high DAPSA, n (%)	76 (30.4)	28 (21.4%)	48 (40.3)	0.007
MDA, n (%)	105 (42.0)	62 (47.3)	43 (36.1)	NS
SCORE2 < 50 = low, n (%)	46 (18.4)	16 (12.2)	30 (25.2)	<0.001
SCORE2 < 50 = moderate, n (%)	15 (6.0)	15 (11.5)	0 (0.0)	<0.001
SCORE2 < 50 = very high, n (%)	2 (0.8)	2 (1.5)	0 (0.0)	NS
SCORE2 50–69 = low-mod, n (%)	75 (30.0)	28 (21.4)	47 (39.5)	<0.001
SCORE2 50–69 = high, n (%)	47 (18.8)	36 (27.5)	11 (9.2)	<0.001
SCORE2 50–69 = very high, n (%)	9 (3.6)	6 (4.6)	3 (2.5)	NS
SCORE2 OP = moderate, n (%)	8 (3.2)	3 (2.3)	5 (4.2)	NS
SCORE2 OP = high, n (%)	21 (8.4)	14 (10.7)	7 (5.9)	0.028
SCORE2 OP = very high, n (%)	8 (3.2)	8 (6.1)	0 (0.0)	0.028
SCORE DM = high, n (%)	7 (2.8)	3 (2.3)	4 (3.4)	NS
SCORE DM = very high, n (%)	12 (4.8)	0 (0.0)	12 (10.1)	NS
Depression, n (%)	46 (18.4)	15 (11.5)	31 (26.1)	0.005
Fibromyalgia, n (%)	28 (11.2)	6 (4.6)	22 (18.5)	0.001
Carotid plaque, n (%)	90 (36.0)	49 (37.4)	41 (34.5)	NS
Femoral plaque, n (%)	157 (62.8)	95 (72.5)	62 (52.1)	0.001
Aortic calcification, n (%)	79 (31.6)	44 (33.6)	35 (29.4)	NS
Obesity, n (%)	56 (22.4)	25 (19.1)	31 (26.1)	NS
Hypertension, n (%)	89 (35.6)	52 (39.7)	37 (31.1)	NS
Diabetes, n (%)	25 (10.0)	9 (6.9)	16 (13.4)	NS
Dyslipidemia, n (%)	169 (67.6)	91 (69.5)	78 (65.5)	NS
Hyperuricemia, n (%)	54 (21.6)	43 (32.8)	11 (9.2)	<0.001

Yrs: years; IQR: interquartile range; PsA: psoriatic arthritis; BMI: body mass index; HDL: high-density lipoprotein; HAQ: health assessment questionnaire; PsAID: PsA impact of disease; cIMT: carotid intima-media thickness; mm: millimeter; DIP: distal interphalangeal joint; IBD: inflammatory bowel disease; CVD: cardiovascular disease; BSA: body surface area; rem: remission; MDA: minimal disease activity; SCORE2: systematic coronary risk evaluation 2; OP: older-people; DM: diabetes mellitus; n: number; NS: non-significant.

## Data Availability

Data supporting the findings of this study are available from the corresponding author upon reasonable request. Due to patient confidentiality and institutional policies, raw individual-level data cannot be made publicly available.

## Supplementary Materials

The following supporting information can be downloaded at: https://www.mdpi.com/article/10.3390/biomedicines14061395/s1. Supplementary Material S1 includes Table S1 (conditional probability matrix) and Table S2 (joint distribution of plaque across all territories). Supplementary Material S2 includes multivariable logistic regression analyses and Figure S1: Adjusted OR forest plots. Supplementary Material S3 includes Table S3: Plaque prevalence by SCORE2 categories within age groups.
