# Supplementary material for: Multisite Atherosclerosis and SCORE2-Based Risk Stratification in Psoriatic Arthritis: A Phenotype-Dependent Role of Vascular Territories
Source: Biomedicines. 2026 Jun 20;14(6):1395. doi: 10.3390/biomedicines14061395 (PMC13296767; doi:10.3390/biomedicines14061395)
Supplement: Supplementary file 1 [file biomedicines-14-01395-s001.zip › biomedicines-4338511-supplementary.pdf]

Supplementary material S1. Conditional probabilities and overlap of atherosclerotic plaques

We evaluated the overlap between vascular territories using conditional probabilities and joint distributions. For each index territory (carotid, femoral, or aortic), we calculated the probability of plaque in the other territories conditional on the presence of plaque in the index. In addition, we estimated the probability of simultaneous involvement of both remaining territories. A joint distribution table was generated to describe the frequency of all possible plaque combinations (000 to 111, representing absence or presence of carotid, femoral, and aortic plaques). This approach allows quantification of concordance patterns and highlights the diagnostic yield of examining multiple vascular beds beyond the carotid arteries alone.

Table S1. Conditional probability matrix

Conditioner ↓ / Target →	Carotid (%)	Femoral (%)	Aortic (%)
Carotid	100.0	88.9	53.3
Femoral	51.0	100.0	47.1
Aortic	60.8	93.7	100.0

Proportion of patients with plaques in both other territories given conditioner plaque:

- Carotid: 52.2%

- Femoral: 29.9%

- Aortic: 59.5%

Table S2. Joint distribution of plaque across all territories

Pattern (Carotid-Femoral-Aortic)	N	%
000	79	31.6
001	4	1.6
010	50	20.0
011	27	10.8
100	9	3.6
101	1	0.4
110	33	13.2
111	47	18.8

Takeaways

- Femoral plaque, when present, co-occurs very frequently with aortic (93.7%) and carotid (88.9%) plaques; but only ~30% of femoral-positive patients have both other beds simultaneously.

- Carotid-positive patients have ~51% femoral and ~61% aortic plaque; 52% have both femoral and aortic.

- Aortic-positive patients have ~53% carotid and ~47% femoral; ~60% have both other beds.

- From the joint table, the most common positive patterns are femoral only (20.0%), carotid+femoral (13.2%), and all three (18.8%); isolated aortic plaque is rare (1.6%).

Supplementary material S2

Covariates included in all models were sex, age, psoriasis duration, arthritis duration, BMI, smoking status (ever vs never smoker), presence of any cardiovascular risk factor (hypertension, diabetes mellitus, dyslipidemia, obesity or hyperuricemia), erosive disease, DAPSA category, PsAID category and biologic exposure. Results are presented as adjusted odds ratios (ORs) with 95% confidence intervals.

Carotid

Variable	OR	95% CI	p-value
Female sex (ref: male)	0.48	0.24–0.98	0.045
Age, per year	1.11	1.07–1.16	<0.001
Psoriasis duration, years	NS	NS	NS
Arthritis duration, years	1.05	1.00–1.11	0.064
BMI, kg/m²	NS	NS	NS
Smoking (ever vs never)	2.55	1.27–5.12	0.009
Any CV risk factor	3.18	1.04–9.69	0.042
Erosions	5.81	2.31–14.59	<0.001
DAPSA moderate–high	NS	NS	NS
PsAID ≥4	2.36	1.07–5.19	0.033
Biologic exposure	5.69	2.37–13.63	<0.001

Femoral

Variable	OR	95% CI	p-value
Female sex (ref: male)	0.14	0.05–0.35	<0.001
Age, per year	1.16	1.10–1.21	<0.001
Psoriasis duration, years	NS	NS	NS
Arthritis duration, years	1.12	1.04–1.21	0.003
BMI, kg/m²	1.12	1.01–1.23	0.029
Smoking (ever vs never)	2.05	0.95–4.44	0.068

Any CV risk factor	6.91	2.20–21.71	<0.001
Erosions	NS	NS	NS
DAPSA moderate–high	3.73	1.35–10.33	0.011
PsAID ≥4	NS	NS	NS
Biologic exposure	NS	NS	NS

Aortic

**Variable**	**OR**	**95% CI**	**p-value**
Female sex (ref: male)	NS	NS	NS
Age, per year	1.09	1.05–1.14	<0.001
Psoriasis duration, years	NS	NS	NS
Arthritis duration, years	1.05	0.99-1.11	0.079
BMI, kg/m²	NS	NS	NS
Smoking (ever vs never)	2.29	1.15–4.56	0.018
Any CV risk factor	9.51	1.98–45.69	0.005
Erosions	NS	NS	NS
DAPSA moderate–high	2.20	0.96–5.03	0.063
PsAID ≥4	NS	NS	NS
Biologic exposure	2.15	0.96–4.81	0.064

Multisite (≥2)

**Variable**	**OR**	**95% CI**	**p-value**
Female sex (ref: male)	0.32	0.15–0.69	0.004
Age, per year	1.17	1.12–1.23	<0.001
Psoriasis duration, years	NS	NS	NS
Arthritis duration, years	NS	NS	NS
BMI, kg/m²	NS	NS	NS
Smoking (ever vs never)	2.24	1.11–4.52	0.024

Any CV risk factor	NS	NS	NS
Erosions	4.27	1.60–11.43	0.004
DAPSA moderate–high	NS	NS	NS
PsAID ≥4	NS	NS	NS
Biologic exposure	4.46	1.86–10.70	<0.001

Figures S1. adjusted OR forest plots (one per outcome):

Forest plots showing adjusted odds ratios (ORs) and 95% confidence intervals derived from multivariable logistic regression models. Covariates included sex, age, psoriasis duration, arthritis duration, body mass index (BMI), smoking status (ever vs never smoker), presence of any cardiovascular risk factor, erosive disease, DAPSA category, PsAID category, and biologic exposure. The vertical reference line indicates OR = 1.

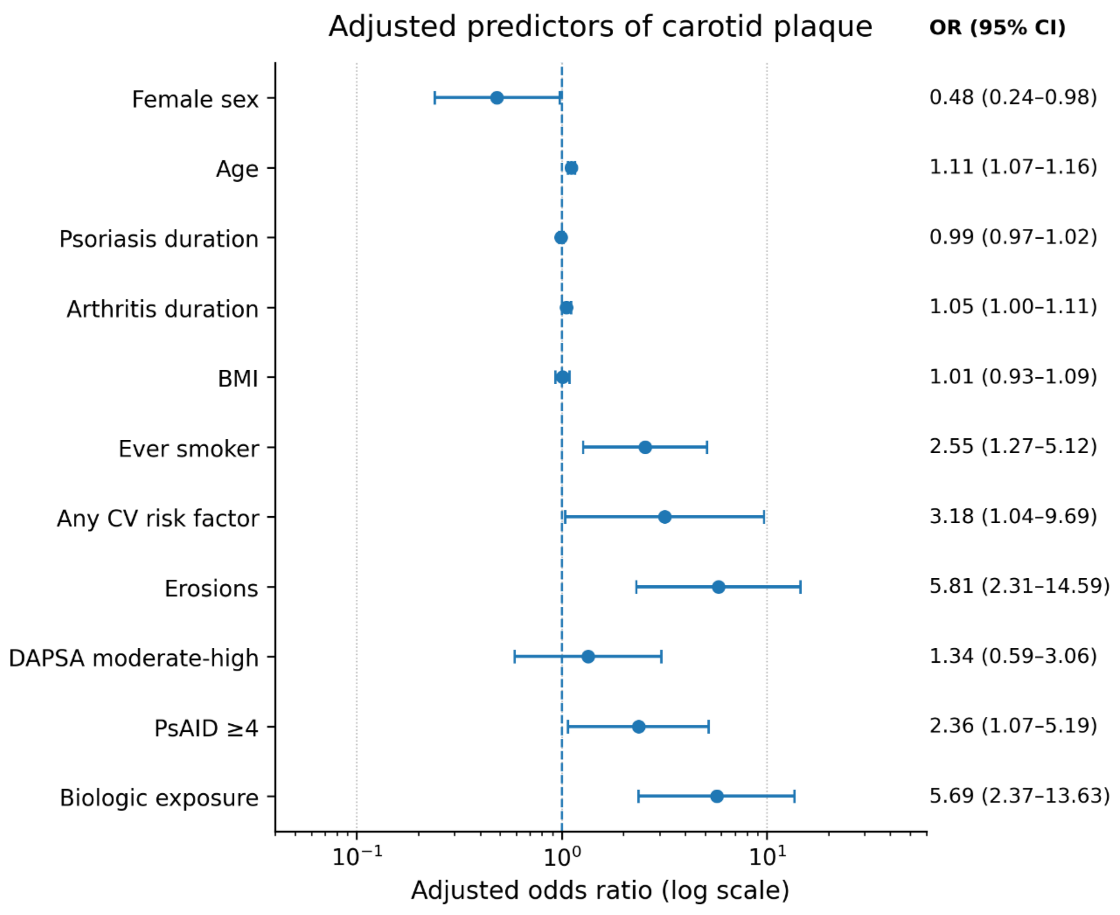

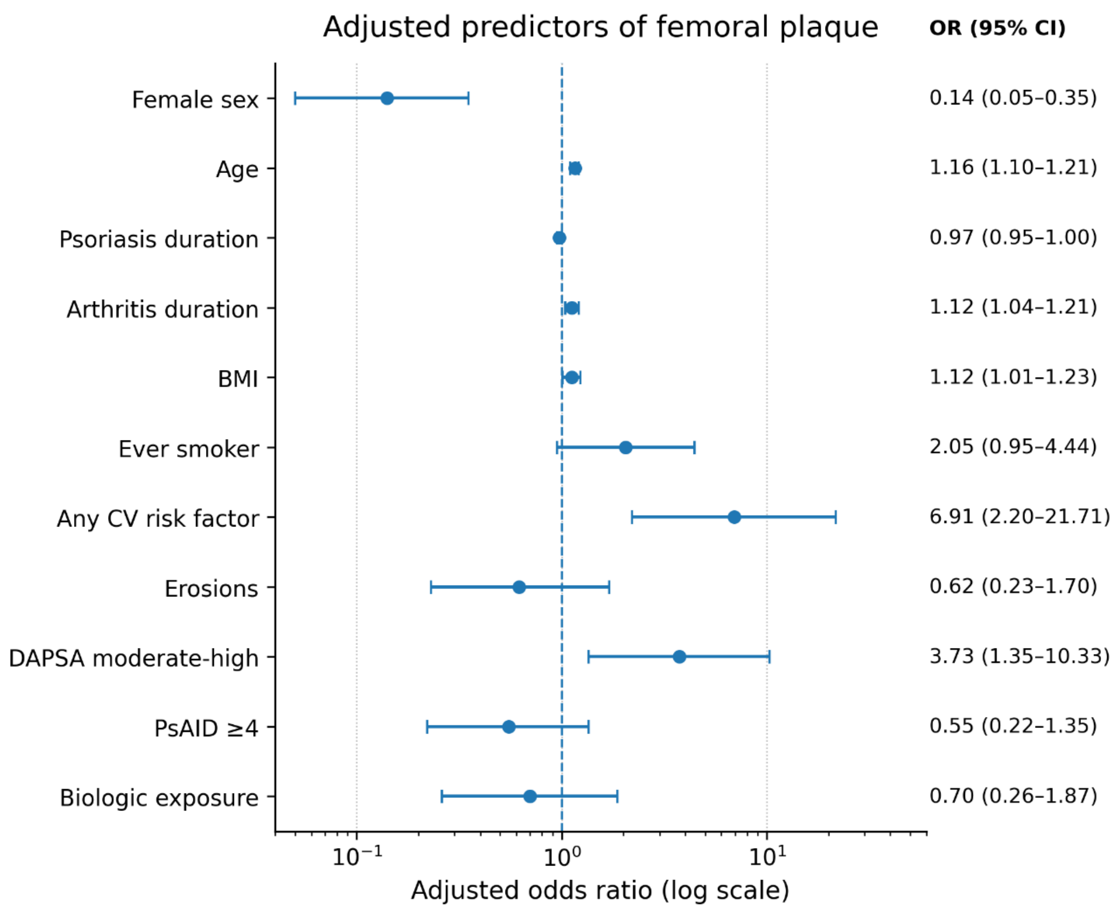
Adjusted predictors of femoral plaque
OR (95% CI)
Female sex
Age
Psoriasis duration
Arthritis duration
BMI
Ever smoker
Any CV risk factor
Erosions
DAPSA moderate-high
PsAID ≥4
Biologic exposure
0.14 (0.05–0.35)
1.16 (1.10–1.21)
0.97 (0.95–1.00)
1.12 (1.04–1.21)
1.12 (1.01–1.23)
2.05 (0.95–4.44)
6.91 (2.20–21.71)
0.62 (0.23–1.70)
3.73 (1.35–10.33)
0.55 (0.22–1.35)
0.70 (0.26–1.87)
10^{-1}
10^{0}
10^{1}
Adjusted odds ratio (log scale)

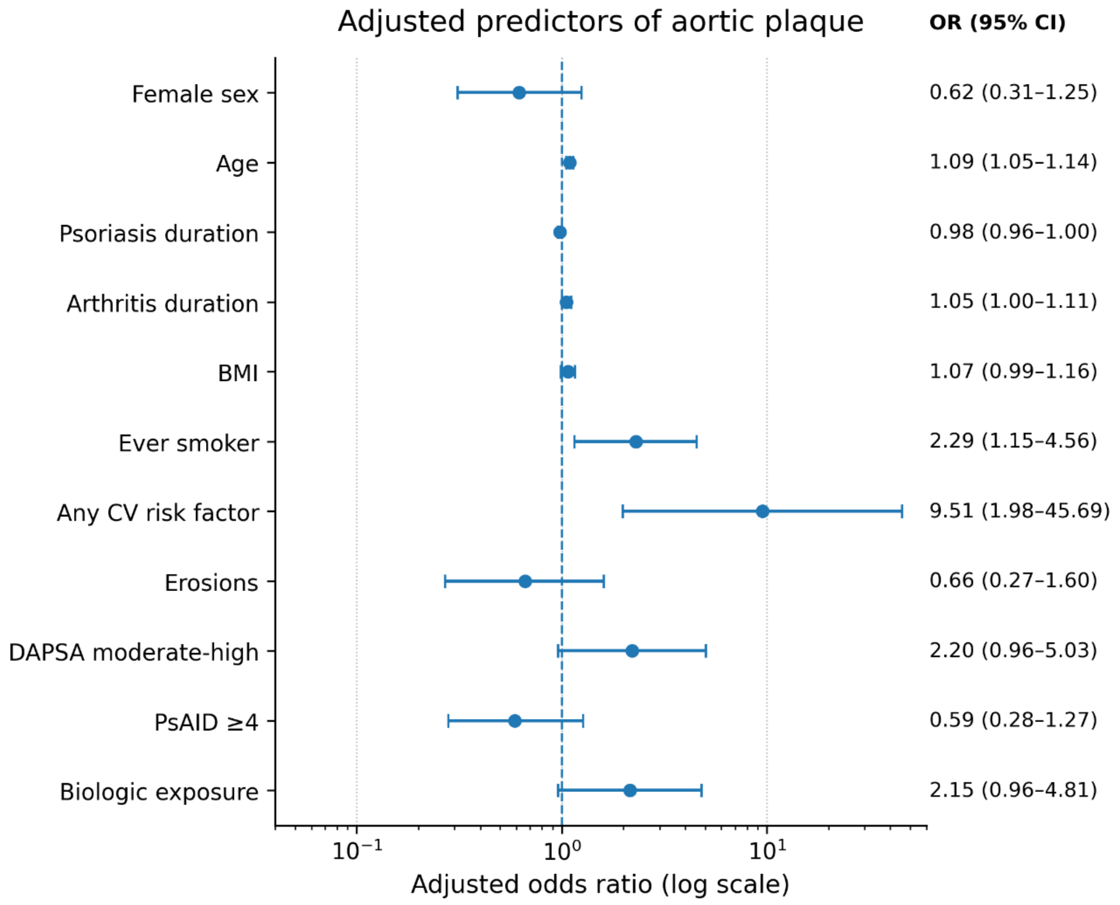
Adjusted predictors of aortic plaque
OR (95% CI)
Female sex
Age
Psoriasis duration
Arthritis duration
BMI
Ever smoker
Any CV risk factor
Erosions
DAPSA moderate-high
PsAID ≥4
Biologic exposure
0.62 (0.31–1.25)
1.09 (1.05–1.14)
0.98 (0.96–1.00)
1.05 (1.00–1.11)
1.07 (0.99–1.16)
2.29 (1.15–4.56)
9.51 (1.98–45.69)
0.66 (0.27–1.60)
2.20 (0.96–5.03)
0.59 (0.28–1.27)
2.15 (0.96–4.81)
10^{-1}
10^{0}
10^{1}
Adjusted odds ratio (log scale)

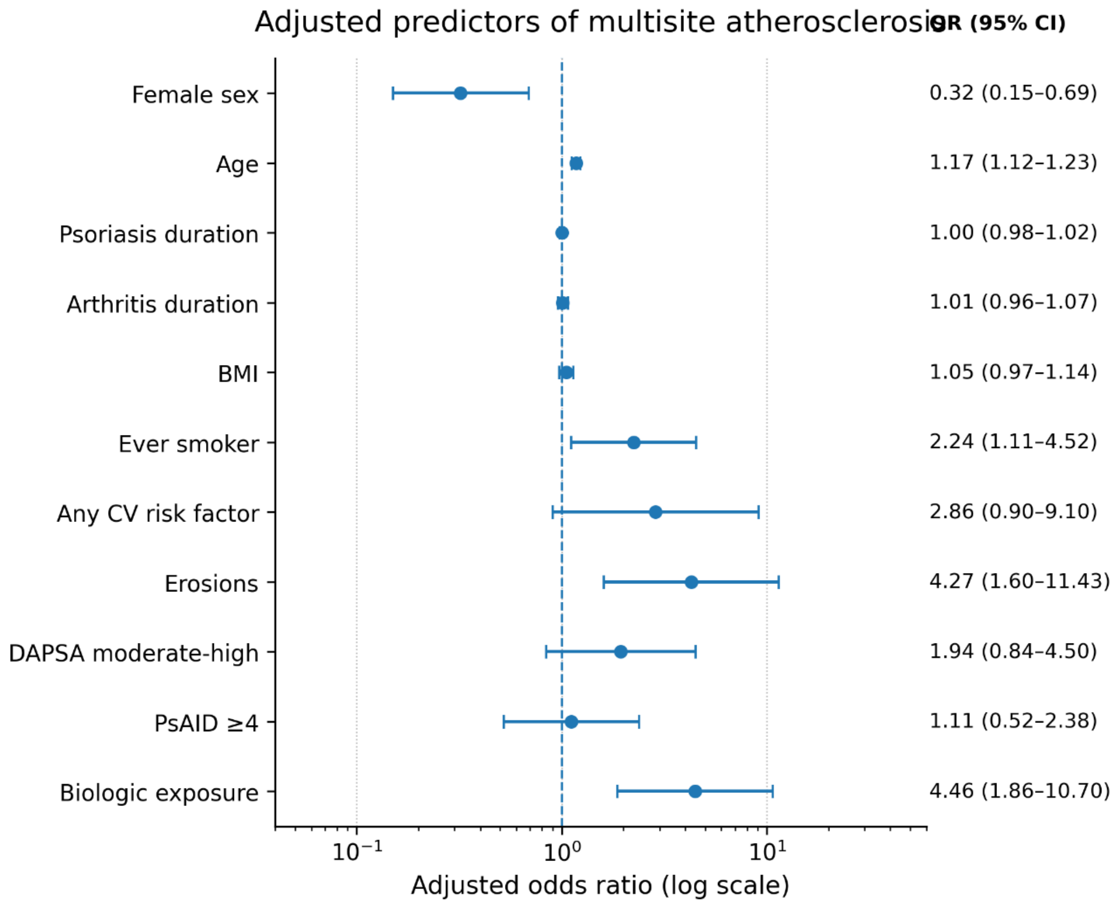

Adjusted predictors of multisite atherosclerosis
OR (95% CI)
Female sex
Age
Psoriasis duration
Arthritis duration
BMI
Ever smoker
Any CV risk factor
Erosions
DAPSA moderate-high
PsAID ≥4
Biologic exposure
0.32 (0.15–0.69)
1.17 (1.12–1.23)
1.00 (0.98–1.02)
1.01 (0.96–1.07)
1.05 (0.97–1.14)
2.24 (1.11–4.52)
2.86 (0.90–9.10)
4.27 (1.60–11.43)
1.94 (0.84–4.50)
1.11 (0.52–2.38)
4.46 (1.86–10.70)
10^{-1}
10^{0}
10^{1}
Adjusted odds ratio (log scale)

Supplementary material S3

Table S3. Plaque prevalence by SCORE2 categories within age groups

Age group	Outcome	SCORE2 category	Prevalence (%)
<50	Carotid	0	4.3
<50	Carotid	1	20.0
<50	Carotid	3	0.0
<50	Femoral	0	13.0
<50	Femoral	1	46.7
<50	Femoral	3	100.0
<50	Aortic	0	0.0
<50	Aortic	1	20.0
<50	Aortic	3	100.0
<50	Multisite (≥2)	0	0.0
<50	Multisite (≥2)	1	20.0
<50	Multisite (≥2)	3	100.0
50–69	Carotid	1	26.7
50–69	Carotid	2	53.2
50–69	Carotid	3	77.8
50–69	Femoral	1	56.0
50–69	Femoral	2	87.2
50–69	Femoral	3	88.9
50–69	Aortic	1	20.0
50–69	Aortic	2	48.9
50–69	Aortic	3	44.4
50–69	Multisite (≥2)	1	25.3
50–69	Multisite (≥2)	2	72.3
50–69	Multisite (≥2)	3	66.7
≥70	Carotid	1	75.0
≥70	Carotid	2	52.4
≥70	Carotid	3	87.5
≥70	Femoral	1	87.5
≥70	Femoral	2	90.5
≥70	Femoral	3	100.0
≥70	Aortic	1	12.5
≥70	Aortic	2	38.1
≥70	Aortic	3	87.5
≥70	Multisite (≥2)	1	75.0
≥70	Multisite (≥2)	2	71.4
≥70	Multisite (≥2)	3	87.5

The table shows the proportion of patients with carotid, femoral, aortic, and multisite (≥2 territories) plaques according to SCORE2 categories, stratified by age group. SCORE2 categories were defined as follows: <50 years (0 = low, 1 = moderate, 2 = high), 50–69 years (1 = low–moderate, 2 = high, 3 = very high), and ≥70 years (1 = moderate, 2 = high, 3 = very high). A clear gradient of increasing plaque prevalence is observed across categories in all age groups, with femoral and multi-site involvement dominating in the high and very high-risk strata**.**

Trend test (chi-square across SCORE2 categories): Carotid: p = 3.398e-09; Femoral: p = 3.465e-16; Aortic: p = 2.086e-10; Multisite (≥2): p = 1.2e-16

Interpretation

- A strong risk gradient is evident: higher SCORE2 category = higher probability of plaque in all territories.
- Femoral plaque is the most frequent at all risk levels, even in “moderate” SCORE2.
- Carotid plaque (often the clinical focus) underestimates systemic disease: only ~30% at moderate risk vs ~57% femoral.
- Multisite involvement becomes dominant from high risk upwards (>70%).
